# School Hygiene—I

**Published:** 1880-04

**Authors:** 


### The Bistoury.

ELMIRA, N. Y., APRIL., 1880.

The BISTOURY is published Quarterly, upon the first of April, July, October and January, at Fifty Cents a year in advance.

### SCHOOL HYGIENE.--I

The public are so slow to respond to any advocacy of a needed reform,—particularly when their physical welfare is concerned, that but little inducement is offered for instructing them in such matters. But, inasmuch as the warnings and pleadings of the clergy, who for years are called upon to preach the same truths to the very same auditors, go unrewarded for indefinite periods of time, perhaps it is expected that the press should hammer away as persistently and uncomplainingly until at last their warnings and recommendations are heeded and acted upon.

Every thoughtful man must have observed the alarming increase of difficulties of vision among the school-children. Voluminous reports have been made by competent oculists in Germany and England, and recently in our own country, showing the causes for this constantly spreading ailment. Dr. Cohn of Breslau, recently examined the eyes of more than ten thousand school children, from which large number it was ascertained that nearly *sixty per cent.* of advanced scholars suffered from nearsightedness, in varying degrees! Further, that the nearsightedness was directly attributable to the faulty construction of the school buildings!

This statement would seem to be startling enough to arrest the attention of the most phlegmatic temperament. Let us consider briefly, the conditions that bring about results so detrimental to our children :

1. Illy constructed school buildings with reference to light.
2. The same with reference to ventilation.
3. Illy adapted school furniture.
4. Badly constructed text-books.—*a* in type; *b.* in breadth of page; *c.* in color of ink and tint of paper.

Other causes could be assigned, such as sending children to school before they have reached an age at which their physical development has been sufficient to sustain them during the trying ordeal of six hours of confinement.[1] But we will confine ourselves to a consideration of the four propositions given, as bearing more directly upon the matter of impaired vision.

First, then: In the present arrangement of school buildings, the light usually comes either from one side, (unilateral,) or from both sides, (bilateral.)

Of course, the light should never come in front, so as to shine in the face of the pupil. No building committee, however obtuse, would think of exposing the eyes to such a glare. Neither should it come from the rear, for then the body interferes with proper illumination.

In unilateral illumination, if to the right, a shadow is cast by the hand in writing or drawing, while both pupils are not equally expanded, the one in the shadow of the nose, on the left, being larger than the right one, thus giving rise to indistinctness of the image in one or the other eye. The scholar, from this cause, is compelled to hold his face nearer his work, a position favoring congestion of the retina and subsequent myopia. If the illumination comes from the left, the same conditions are present, only being a more favorable light for writing or drawing.

Bilateral illumination is less detrimental than unilateral, but is objectionable because of the unequal quantity of light obtained for each eye, particularly by those sitting next the windows. If the room is large, the scholars seated in the middle have insufficient light, requiring them to bring their books too close to their faces.[2] Then, the windows run too near the floor, bringing the light too full upon the face. It were better if the bottom of the window came above the pupils' head, and extended from thence to the very ceiling.

The very best light would be that obtained from a large glass dome on top the building, aided by windows in the walls to the right and left of the scholars, far above their heads. These lateral windows should be properly fitted with shades to exclude direct sunlight, and with cords for opening and closing them to regulate ventilation.

All our school buildings, therefore, should

1. A child should never, under any circumstances, be sent to a regular school until six or seven years of age. He should have the utmost liberty to run and play, until that period.

2. A room 20 ft. square should not contain less than 80 square feet of glass. Indeed, too much light cannot be afforded any school room where proper attention is paid to excluding all glare by means of frequently adjusted curtains.

be but one story high and spread over the ground instead of being projected into the air. By the arrangement of the windows near the ceiling, and from the dome on the roof, abundant diffused illumination is obtained, without having a flood of light fall full upon the face.

A building so constructed would have other advantages. It would be cool in summer and warm in winter. The attention of the scholars would not be distracted from their lessons by what is transpiring outside, for no view of outdoors could be obtained. There would exist less liability to accident in having scholars thrown down stairs in the scufflings of boys, or in the excitement incident to fire and other alarms. Such buildings would be more expensive in our largest cities, by reason of the valuable ground they occupy, but the matter of expense will not weigh against the endeavor to protect the sight and health of our children.

Under the second head, we might note the many excellent arrangements that have been suggested by numerous writers upon the subject. It is a problem very difficult of solution in a climate like ours, where warmth is quite as essential as pure air. It will not answer to open windows and doors, subjecting the children to drafts of air and causing their bodies to shiver with cold while their brains are recovering from the depressing influences of noxious gases. We have only to enter any of our well-filled school rooms to be oppressed by the exhalations from the bodies and lungs of the inmates, and to be convinced of the urgent necessity for a change in the methods of ventilation.

Bad air has a direct influence upon the eyes, often causing a congestion of its mucous surfaces that leads to a more serious inflammation. Even more grave is its deleterious consequences upon the general health, a train of evils thus originated that we cannot stop to enumerate. The remedy lies with the architect of the building. No edifice should be constructed without the advice of a skillful architect upon the proper means for ventilation. It is a matter not to be entrusted to a mere tyro, but to one thoronghly versed in his profession.

Upon the the third head it must be said that our school furniture is faulty in construction, in that it is not adjustable to the size of the pupil. The desk should not be so low as to cause the scholar to bend over it, so inviting an undue flow of blood to the head. Neither should it be too high, so inconveniencing him in writing. Either fault is likely to work injury to the body as well as to the eye. If the seat is too high, giving no support to the body from the feet, spinal curvatures are likely to be invited, while the scholar is made weary from his unnatural position.

The only remedy we can see for this difficulty is in having the desks and sittings made adjustable, so that they can be made higher or lower to suit every individual scholar. This does not seem to be a very difficult matter to arrange, as it could be easily and inexpensively accomplished by a cog and ratchet arrangement fitted to the standards of the desks and stools, that lowering or elevating would be permitted by means of a crank. (This hint is offered gratuitously to manufacturers of school furniture whom we would like to see act upon it.)

The fourth head is equally important, although not so readily recognized by the casual observer.

The type should never be fine, as used in poetry, foot notes, and the like. The type in which this journal is printed, is too fine for text books. What is known as long primer is the proper size and should be of a bold, full face. If finer, the book is brought too close to the eyes. Young children should never bring the book nearer than twelve inches of the face. Adults should be enabled to read it readily at from 13 to 15 inches. Much nearer than this indicates faulty books or imperfect eyes.

The page should always be in double column. If the line extends entirely across the page, it wearies the muscles of the eyes in constantly directing them laterally toward each end of the long line, besides often leading to confusion and increased effort to find the next succeeding line. This seems like a slender objection, but every oculist will agree in that its weakening power upon the eyes is not inconsiderable.

It is also important that the ink should be black, never pale, and never in colors. The paper should not be white and glistening, but of a pale yellow tint. The paper upon which this journal is printed is of the tint recommended as being most agreeable to the eye. Highly finished papers, with a polished surface, reflect a glistening light that is very disagreeable to the eye.

It is important also that the space between the lines should not be too small nor too great. In the first instance, it will cause confusion, in the latter, fatigue. The distance from the bottom of one line to the top of the succeeding one is found to be most agreeable to the eyes when one-eighth of an inch apart.

If parents could be made to see the great importance of the reforms recommended, or better still, if the boards of education would consider them in the building of school houses and in the adoption of school furniture and school books, a great benefit would accrue to our children.

In the examination of 2,200 school children in and around Bern, Switzerland, the tables of which we have before us, it is shown that the vast majority of children begin school life with perfectly normal eyes. As they are advanced in their studies, myopia increases. In the primary schools, at the end of the first year, 6.7 per cent. are nearsighted. In the intermediate grade 10.3 per cent. In the high schools 19.7 per cent. In the colleges 26.2 per cent, while in some of the higher classes *sixty per cent.* of the pupils were found to be myopic.

If any evidence were needed to substantiate the fact that faulty buildings was the direct cause of this alarming condition of affairs, it will be found in the circumstance that in the rural schools,) the buildings of which were not surrounded by brick walls—as is usually the case in the larger cities—the rooms smaller, the curriculum less severe and the arrangement of light better,) cases of near-sightedness was extremely rare.

It seems difficult to make parents comprehend that a near-sighted eye is a *diseased* eye. Many are given to the opinion that such eyes are really superior to normal ones—that they are "stronger," and the opinion seems to be universal, that the happy possessors will be able to dispense with spectacles when they reach the age that old folks are required to seek these aids to vision. Nothing could be further from the truth. Not only are near-sighted eyes *always* required to wear spectacles but usually stronger and stronger ones, the bulging of the back of the eye increasing often to the extent of producing blindness.

With these cautionary words we will dismiss the subject for the present to resume it next quarter, when we will have something to say of school discipline and its effect upon the eyes.

